# Electrochemical Properties of Mo_4_VC_4_T_*x*_ MXene in Aqueous Electrolytes

**DOI:** 10.1021/acsami.4c06519

**Published:** 2024-07-15

**Authors:** Iftikhar Hussain, Faisal Rehman, Mohit Saraf, Teng Zhang, Ruocun Wang, Tridip Das, Zhengtang Luo, Yury Gogotsi, Kaili Zhang

**Affiliations:** † Department of Mechanical Engineering, City University of Hong Kong, 83 Tat Chee Avenue, Kowloon, Hong Kong 19104, China; ‡ A.J. Drexel Nanomaterials Institute and Department of Materials Science and Engineering, Drexel University, Philadelphia, Pennsylvania 19104, United States; § Department of Chemical and Biological Engineering, The Hong Kong University of Science and Technology, Clear Water Bay, Kowloon, Hong Kong19104,China; ∥ Materials and Process Simulation Center (MSC), MC 139-74, California Institute of Technology, Pasadena, California 91125, United States; ⊥ Department of Chemical & Polymer Engineering, University of Engineering & Technology Lahore, Faisalabad Campus, 3.5km, Khurrianwala − Makkuana By-Pass, Faisalabad 38000, Pakistan

**Keywords:** Mo_4_VC_4_T_*x*_ MXene, DFT, AIMD simulation, aqueous electrolytes, supercapacitors

## Abstract

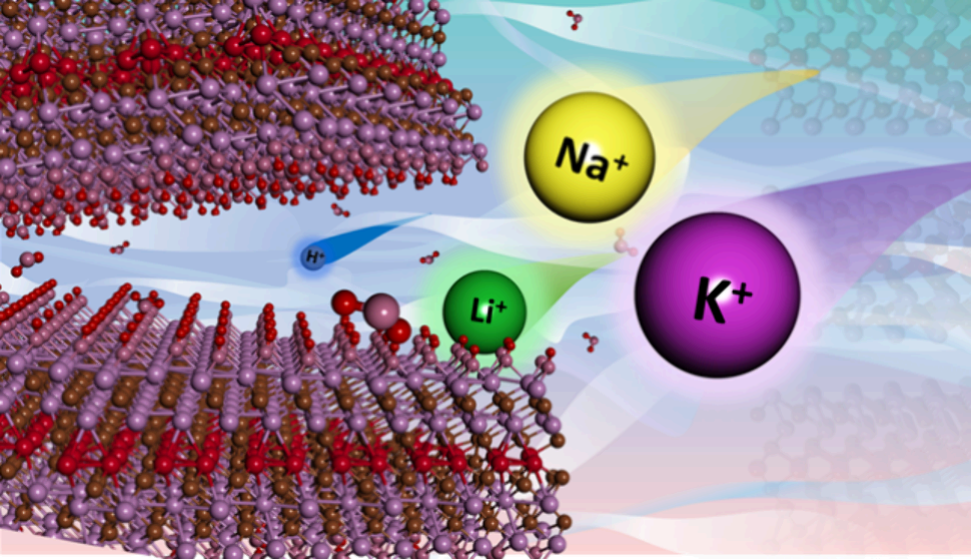

M_5_C_4_T_*x*_ MXenes represent the most recently discovered and least studied subfamily of out-of-plane ordered double transition metal carbides with 11 atomic layers, probably the thickest of all 2D materials. Molybdenum (Mo) and vanadium (V) in Mo_4_VC_4_T_*x*_ offer multiple oxidation states, making this MXene potentially attractive for electrochemical energy storage applications. Herein, we evaluated the electrochemical properties of Mo_4_VC_4_T_*x*_ free-standing thin films in acidic, basic, and neutral aqueous electrolytes and observed the highest gravimetric capacitance of 219 F g^–1^ at 2 mV s^–1^ in a 3 M H_2_SO_4_. Further, we investigated the intercalation states of four different cations (H^+^, Li^+^, Na^+^, and K^+^) in MXenes through *ab initio* molecular dynamics (AIMD) simulation and used density functional theory (DFT) calculations to assess the charge storage mechanisms in different electrolytes. These studies show hydrated Li^+^, Na^+^, and K^+^ ions forming an electric double layer (EDL) at the MXene surface as the primary charge storage mechanism. This work shows the promise of Mo_4_VC_4_T_*x*_ MXene for energy storage in aqueous electrolytes.

## 1 Introduction

Two-dimensional (2D) materials, such as graphene, hexagonal boron nitride, and transition metal dichalcogenides, have attracted interest owing to their photonic, electrical, optical, and electrochemical properties suitable for a variety of applications.^1,2^ A new range of opportunities for 2D materials was opened by the discovery of MXenes.^3^ The general formula for MXene is M_*n+1*_X_*n*_T_*x*_, where M is a transition metal, X is C or N, *n* = 1–4, and T_*x*_ is the surface termination.^4^ Typical solution-based preparation techniques generate T_*x*_ with a nonuniform mixture of functional groups, such as −OH, −F, and −O.^5^ MXenes have become a major interest in different research fields, including electrochemistry, electromagnetic wave absorption/shielding, catalysis, sensing, biomedicine, energy harvesting, and so on.^6,7^ Over 40 discrete stoichiometric MXenes have been prepared, including Ti_3_C_2_T_*x*_, V_2_CT_*x*_, V_4_C_3_T_*x*_, and Nb_2_CT_*x*_, as well as numerous solid solutions. Hundreds more are predicted to be thermodynamically stable, and an infinite number of solid solutions and chemically terminated MXene can be created.^8^

MXenes have a high electrical conductivity that facilitates electron transport inside 2D structures^9^ and 2D slits between the flakes allow rapid ion diffusion, which is beneficial for electrodes in energy storage devices. Vanadium-^10,11^ and molybdenum-based^12,13^ MXenes have exhibited excellent performance in batteries and supercapacitors due to reversible redox reactions of those transition metals. MXenes have shown electric-double layer capacitive (EDLC) and pseudocapacitive storage mechanisms in acidic, basic, and neutral electrolytes.^14−17^ Incorporating a secondary transition metal into MXenes with desirable characteristics might improve their electrochemical properties. Recently, Mo-based ordered double-transition-metal carbide MXene Mo_2_Ti_2_C_3_ has been explored in supercapacitors.^18,19^

M_5_C_4_T_*x*_ MXenes represent the most recent and least studied subfamily of double transition metal carbides with arguably the thickest layers among all 2D materials. The first discovered composition was Mo_4_VC_4_T_*x*_,^20^ and a couple of other compositions in this family followed,^21^ potentially offering attractive optical, electronic, and thermal properties. They have an unusual, twinned structure, which differentiates them from other MXenes. The increased number of atomic layers in MXenes presents opportunities for enhanced mechanical performance, better EMI shielding, and improved electrical conductivity in various applications. However, their electrochemistry and energy storage applications have yet to be explored. Mo_4_VC_4_T_*x*_ MXenes can offer multiple oxidation states of Mo and V, as well as redox activity in a variety of electrolytes, which may enable pseudocapacitive charge storage properties.

In this work, we prepared Mo_4_VC_4_T_*x*_ and combined an experimental study with multiscale simulations/modeling to investigate the interaction of cations with water molecules and MXene surfaces under confinement. *Ab initio* molecular dynamics (AIMD) calculations were used because of their high accuracy in determining the preferred positions of protons and the monovalent Li^+^, Na^+^, and K^+^ cations within the confinement of MXene and the surrounding hydrated environment. The intercalation of electrolyte ions was also studied through projected density of states (PDOS) calculations. Electrochemical responses of the prepared free-standing electrodes of Mo_4_VC_4_T_*x*_ MXenes were evaluated in acidic, neutral, and basic electrolytes. The results indicate that the potential window of electrochemical stability of Mo_4_VC_4_T_*x*_ depends on the electrolyte: −1.0 V to −0.5 V in KOH, −0.8 V to −0.1 V in Na_2_SO_4_, −0.8 to 0.2 V in LiCl, and −0.25 to 0.3 V in H_2_SO_4_.

## 2 Experimental Section

### 2.1 Synthesis of Mo_4_VAlC_4_ MAX and Free-Standing Mo_4_VC_4_T_*x*_ MXene Film

The synthesis of Mo_4_VAlC_4_ was carried out based on our earlier work.^20^ Using an agate mortar and pestle, powders of molybdenum (99.9% Alfa Aesar, 250 mesh), vanadium(III) oxide (98% Sigma-Aldrich), vanadium (99.5% Alfa Aesar, 325 mesh), aluminum (99.5% Alfa Aesar, 325 mesh), and graphite (99% Alfa Aesar, 325 mesh) – Mo (4): V_2_O_3_ (0.05): V (0.9): Al (1.2): C (3.5), were ball-milled for 90 min. The powder mixtures were heated in a tube furnace (Carbolite Gero) at a 3 °C/min rate under 350 cm^3^/min of flowing argon until it reached 1650 °C. After holding the temperature (1650 °C) for 4 h, the furnace was allowed to cool naturally to ambient temperature. The resulting sintered Mo_4_VAlC_4_ MAX phase blocks were carefully removed and drilled into powder using a tabletop drill press with a carbide drill bit. To further remove its impurities, 15 g of the Mo_4_VAlC_4_ MAX powder was agitated in 50 mL of HCl (36.5–38% Fisher Chemical) for 18 h to dissolve the metallic and oxide impurities. The HCl was removed from the mixture through centrifugations at 3500 rpm (2550 rcf) for 3 min, decanting the acidic supernatant and redistributing the remaining sediment in DI water. The resulting Mo_4_VAlC_4_ MAX powder was dried for 18 h at 25 °C in a vacuum desiccator. Finally, the Mo_4_VAlC_4_ MAX powder was sieved to less than 75 μm particle size. MXene was prepared by carefully adding MAX powder (2 g) to HF (20 mL, 50% Arcos Organics). The solution was heated in an oil bath at 55 °C for 8 days under continuous stirring using a polytetrafluoroethylene (PTFE)-covered stir bar at 400 rpm. The mixture was then washed with DI water using a series of centrifugations until the pH > 6. The remaining sediment was redispersed and exfoliated in 20 mL of 5 wt % tetramethylammonium hydroxide (TMAOH, 25 wt % - diluted to 5 wt %, Sigma-Aldrich) and further stirred at 400 rpm at 25 °C. After 24 h, the TMAOH was then removed using an additional set of washing cycles. Briefly, the solution was first centrifuged for 10 min at 10,000 rpm. The alkaline supernatant was decanted, and the sediment was redispersed with DI water. The washing was repeated 4 more times but centrifuged for 30 min every time. Due to the stability of the Mo_4_VC_4_ flakes in the alkaline solution, high-speed centrifugation was required. After reaching a pH < 8 for the decanted supernatant, the leftover sediment was redispersed in DI water (30 mL) and bath-sonicated (100 W, 40 kHz) for 1 h with argon bubbling. Then, the solution was centrifuged for an h at 3500 rpm (2550 rcf). To prevent contamination and redistribution between the multilayer MXene and MAX phase sediment, the resulting supernatant was carefully extracted using a pipet and put into a separate new bottle. The colloid comprising the delaminated MXene flakes was filtered using vacuum-assisted filtration through a Celgard 3501 membrane to obtain free-standing film of Mo_4_VC_4_T_*x*_ MXene. The resulting MXene films were then dried for 18 h at 25 °C in a vacuum desiccator and used for further characterization and electrochemical analyses.

### 2.2 Structural and Compositional Characterization

The crystal structure was characterized through Rigaku SmartLab with Ni-filtered Cu–K radiation applied at 40 kV/30 mA. For the as-produced free-standing film of Mo_4_VC_4_T_*x*_, the step size of the scan was 0.01°, and the step duration was set as 4 s. SEM images were produced using FEI Strata DB235 Dual Beam Focused Ion Beam SEM and a Zeiss Supra 50VP scanning electron microscope. The chemical compositions were determined through XPS. A monochromatic Al Kα X-ray source with a 200 μm spot size was used with a spectrometer (Physical Electronics, Versa Probe 5000, MN) to gather XPS spectra. A dual-beam charge neutralizer was used to neutralize the charges within the sample. A pass energy of 23.5 eV with a step size of 0.05 eV was used to collect high-resolution spectra, while a pass energy of 117 eV with a step size of 0.5 eV was used to gather survey spectra. Using Casa XPS software with a linear-type background, the core-level spectra were quantified and fitted with peaks. Raman spectra were acquired using a Renishaw (2008, Gloucestershire, UK) dispersive instrument with 1800 line/mm grating in an inverted reflection mode using a 63× (NA = 0.7) objective. The excitation wavelength was 514 nm, and the laser’s power was maintained between 0.5 and 1 mW.

### 2.3 Electrochemical Properties

A three-electrode cell was prepared using FA Swagelok cells with glassy carbon electrodes acting as current collectors. Working electrodes were made directly from the free-standing MXene film. The counter electrode was made from 95 wt % activated carbon (YP-50) and 5 wt % PTFE. Celgard 3501 was used as a separator, and Ag/AgCl was used as a reference electrode. All electrochemical cells underwent 50 precycles at 20 mV s^–1^. CV measurements were performed from 2 to 1000 mV s^–1^ scan rates. The stability of the electrode was studied at a current density of 10 A g^–1^ for 8000 cycles. The capacitance of the MXene electrodes was determined from the cathodic portion of the CV curves at various scan rates based on the following equation:^22^
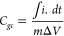
1where *C* is the gravimetric capacitance (F/g), *i* is the current changed by time (*t*), *m* is the mass of the MXene electrode (g), and Δ*V* is the potential window (V). The volumetric capacitance is obtained by multiplying C_*gc*_ by the density of the MXene electrode.

### 2.4 Computational Details

The *ab initio* molecular dynamics simulation was performed and implemented in VASP (5.4.4).^23,24^ For structural optimization, the cutoff energy was set to 450 eV. The structure optimization criteria were set to 10^–6^ eV for energy and 0.02 eV/Å for forces on each atom. We used a 1 × 1 × 1 Gamma-centered k-point grid to sample the Brilliouin zone. The AIMD simulations were performed in the NPT (N = constant number of atoms, P = constant ambient pressure, and T = constant temperature) ensemble, with a time step of 1 fs using the Langevin thermostat. The structural model contains 36 atoms with in-plane lattice parameters *a, b* = 6 Å, and *c* = 18 Å. The MXene layer was supposed to be covered with a bilayer of water molecules. We treated the inserted cations as neutral atoms. The data after 10 *p*s of molecular dynamics was used for the analysis and the number of cations was kept constant for all calculations for better comparison.

## 3 Results and Discussion

**Figure 1:**
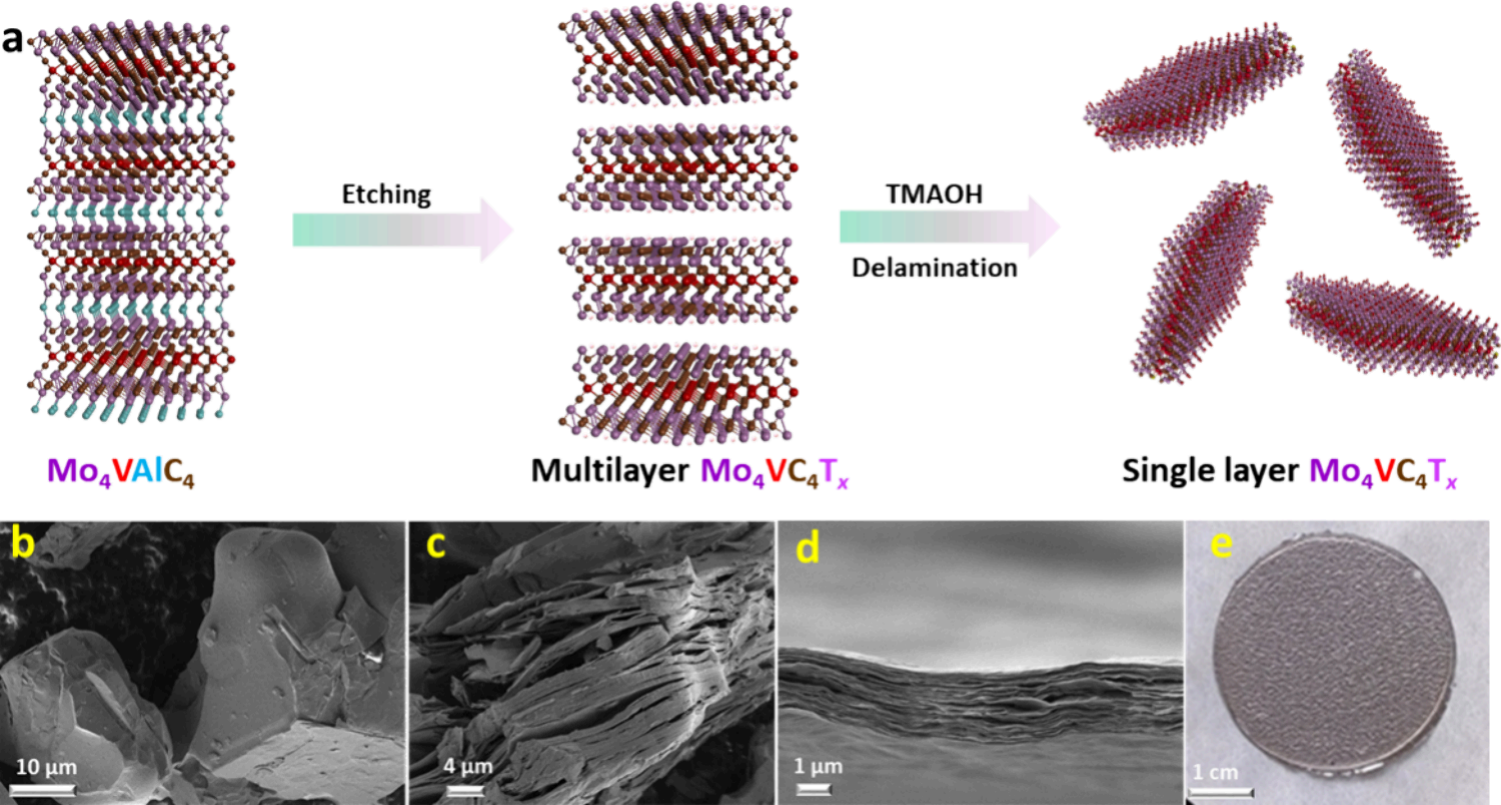
(a) The synthesis of Mo_4_VC_4_T_*x*_ MXene from the Mo_4_VAlC_4_ MAX phase; (b-d) Microscopic analysis of MAX and MXene. SEM micrograph of (b) parent Mo_4_VAlC_4_ MAX phase powder; (c) Multilayer Mo_4_VC_4_T_*x*_ MXene particle, and (d) cross-section of delaminated Mo_4_VC_4_T_*x*_ film. (e) Optical image of the Mo_4_VC_4_T_*x*_ film prepared by vacuum-assisted filtration.

The previously described HF-etching procedure was used to prepare the Mo_4_VC_4_T_*x*_ MXene.^20^ The synthesis approach is illustrated in Figure 1a. Initially, HF acid selectively removes the Al layers of the MAX structure, forming AlF_3_ alongside the surface terminations bonded to the basal planes of the resulting MXene. MXene flakes held together by the weak van der Waals forces were delaminated by introducing tetramethylammonium (TMA^+^) ions, which intercalate between the layers, forcing them apart. Mechanical agitation results in a colloidal suspension of delaminated 2D single-layer Mo_4_VC_4_T_*x*_ flakes.

The scanning electron microscopy (SEM) images of the parent Mo_4_VAlC_4_ are illustrated in Figure 1b. During the HF etching of the MAX, weakly bonded multilayered MXene (Figure 1c) was produced. M-A bonds, which are weaker than M-X bonds, were cleaved during A atomic layer etching, resulting in undercoordinated M metallic surfaces that quickly saturated again by interacting with T_*x*_ species from the etchant.^25^ The MXene multilayer particles were delaminated with TMA^+^ ions to obtain single- or few-layer Mo_4_VC_4_T_*x*_ sheets. Figure 1d exhibits the SEM image of a free-standing film based on a delaminated Mo_4_VC_4_T_*x*_ MXene prepared by vacuum-assisted filtration wherein binders and current collectors were not needed. The average thickness of the as-prepared film was approximately 2 μm. The optical image of the free-standing Mo_4_VC_4_T_*x*_ film is shown in Figure 1e.

**Figure 2:**
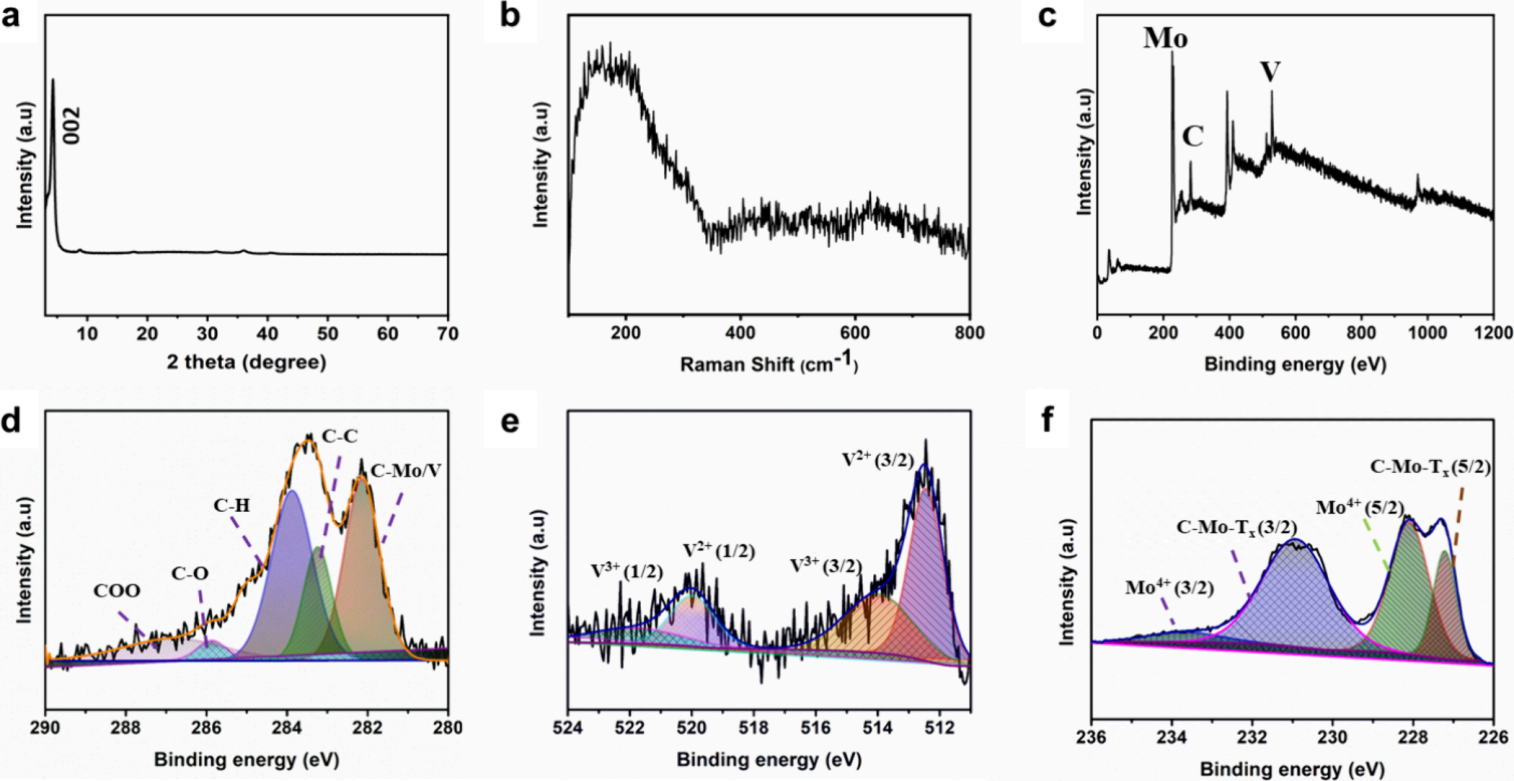
Characterization of Mo_4_VC_4_T_*x*_ MXene: (a) XRD pattern; (b) Raman spectrum; and XPS spectra of Mo_4_VC_4_T_*x*_; (c) survey; (d) C 1s; (e) V 2p; and (f) Mo 3d.

X-ray diffraction (XRD) was used to determine the crystal structure of the delaminated Mo_4_VC_4_T_*x*_ film (Figure 2a). The (002) peak detected in the delaminated Mo_4_VC_4_T_*x*_ film shifted toward a lower 2θ compared with the peak in the multilayer MXene. The peak shift was due to the intercalation of tetramethylammonium cations within the layered structure,^20^ suggesting an increase in the *c* lattice parameter. Other MXenes, including Ti_3_C_2_T_*x*,_ showed comparable increases in *c* lattice parameters upon the completion of delamination.^5^ The Raman spectrum of Mo_4_VC_4_T_*x*_ had broad peaks below 1000 cm^–1^, which were associated with the vibrations of metals with carbon and oxygen (Figure 2b). The Raman spectrum of Mo_4_VC_4_T_*x*_ MXene is similar to the one reported by Deysher et al.^20^ It possesses three regions of vibrations. V, Mo, C, and surface group vibrations were observed between 100 and 300 cm^–1^, in the surface group region between 350 and 500 cm^–1^_,_ and the carbon vibration region between 500 and 700 cm^–1^. Notably, the peaks are broad and overlapped (Figure 2b) like those observed in the earlier study.^20^ The intermixing of Mo and V in the M-layers may be responsible for the broadened Raman peaks.^20,26^

X-ray photoelectron spectroscopy (XPS) survey spectrum (Figure 2c) of Mo_4_VC_4_T_*x*_ confirmed the presence of Mo, V, and C. The C 1s (Figure 2d) was deconvoluted in five different peaks, which were associated with C–Mo/V (∼281.9 eV), C–C (∼283.56 eV), C–H (∼284.11 eV), C–O (∼286.01 eV), and C–OO (∼288.12 eV). As shown in Figure 2e, the V 2p high-resolution spectrum was fitted in two doublets with binding energy centering at ∼513.38 eV (∼520.84 eV) and ∼514.94 eV (∼522.81 eV), which corresponded to the V^2+^ and V^3+^ states, respectively. The two doublets of V 2p in the MXene structure were assigned to the bond between V and C atoms. Moreover, two doublets in the Mo 3d region corresponded to Mo bonded to carbon and surface termination (C–Mo-T_*x*_) at ∼227.47 eV (∼231.68 eV) and Mo^4+^ oxidation state at ∼228.59 eV (∼233.83 eV), which was associated with a negligible amount of oxides present in Mo_4_VC_4_T_*x*_ (Figure 2f).

**Figure 3:**
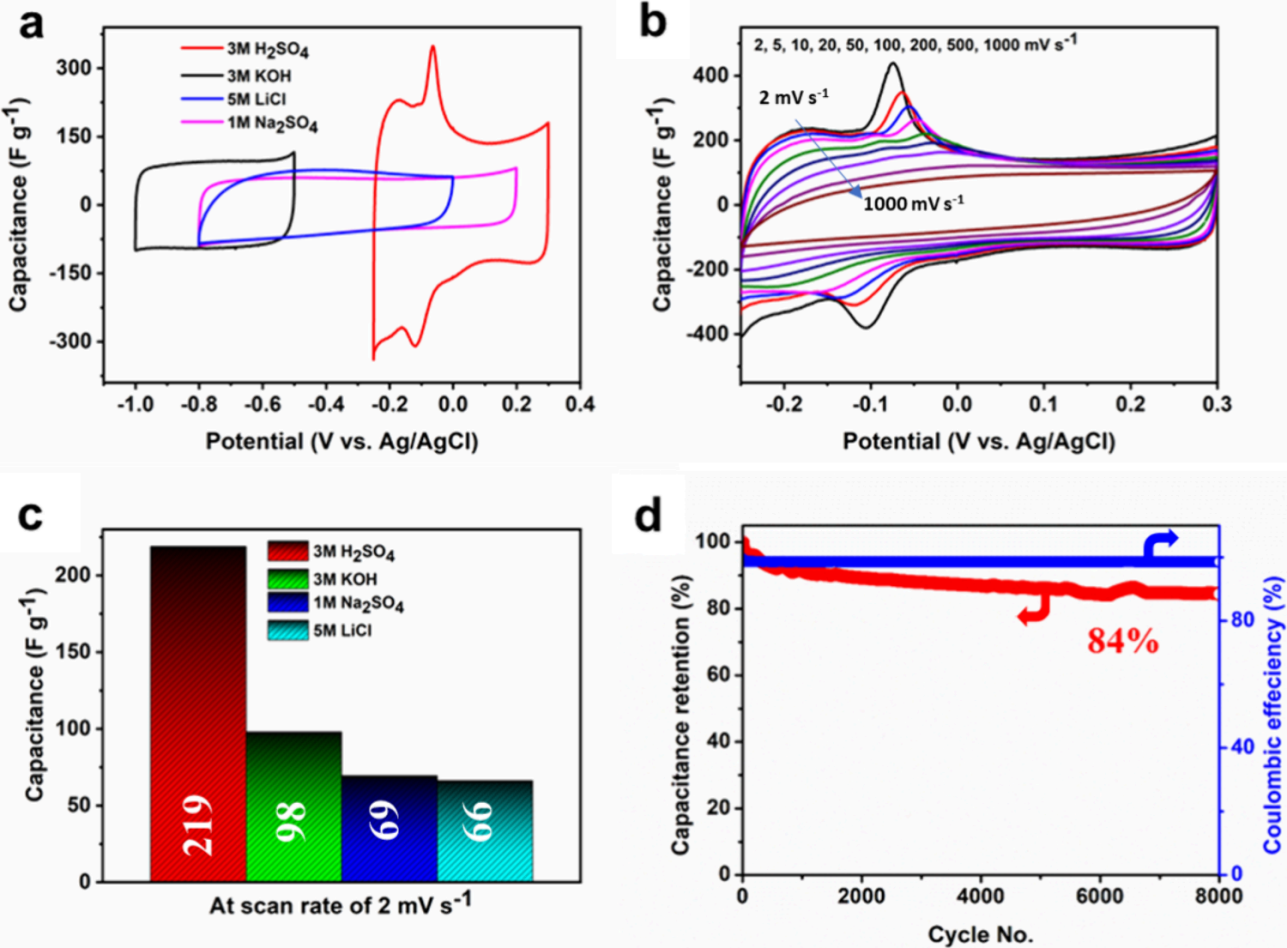
Electrochemical performance of Mo_4_VC_4_T_*x*_ in various electrolytes: (a) CV curves at a scan rate of 5 mV s^–1^ in different electrolytes; (b) CV curves at different scan rates ranging from 2 to 1000 mV s^–1^ in 3 M H_2_SO_4_; (c) specific capacitance at a scan rate of 2 mV s^–1^ in various electrolytes; and (d) cycling stability and Coulombic efficiency in 3 M H_2_SO_4_.

The electrochemical performance of the free-standing Mo_4_VC_4_T_*x*_ electrodes was evaluated using a three-electrode setup in the potential window range of −1.0 to −0.5 V in 3 M KOH, −0.8 to −0.1 V in 1 M Na_2_SO_4_, −0.8 to 0.2 V in 5 M LiCl, and −0.25 to 0.3 V in 3 M H_2_SO_4_, all vs. Ag/AgCl. Comparison of the CV curves of Mo_4_VC_4_T_*x*_ in acidic, neutral, and basic electrolytes at the same scan rate (Figure 3a) indicated a pair of broad redox peaks in an acidic medium, a cathodic peak at approximately −0.11 V (vs Ag/AgCl), and an anodic peak at approximately −0.09 V (vs Ag/AgCl), indicating the contribution of pseudocapacitance. Such pseudocapacitive behavior has also been observed in Ti-based MXene/H_2_SO_4_ systems.^27,28^ In basic and neutral electrolytes, unlike in the acidic electrolyte (Figure S1a), nearly rectangular CV curves without any sign of a redox peak were observed, suggesting that their charging mechanisms were mainly based on double-layer capacitance (Figures S1b–d). Herein, the insertion and deinsertion of ions were responsible for the variation in capacitance behavior.^29^ The detailed CV curves at different scan rates for the Mo_4_VC_4_T_*x*_ MXene films in acidic (Figure 3b), neutral, and basic electrolytes are shown in Figure S1. Among all electrolytes, Mo_4_VC_4_T_*x*_ exhibited the highest specific capacitance (219 F g^–1^) at 2 mV s^–1^ in 3 M H_2_SO_4_ (Figure 3c) and showed peaks that may correspond to protonation/deprotonation of the MXene surface.^30^ The maximum specific capacitance obtained in other electrolytes at the same scan rate of 2 mV s^–1^ was 98 F g^–1^ in 3 M KOH, 69 F g^–1^ in 3 M Na_2_SO_4_, and 66 F g^–1^ in 5 M LiCl. Typical for double-layer capacitance rectangular CVs were observed in neutral and basic electrolytes. Differences in performance among the electrolytes may be due to the varied cations, different charging mechanisms, and other factors, such as solvation shell, ion size, and desolvation energy.^31,32^ Galvanostatic charge–discharge (GCD) profiles (Figure S2) of Mo_4_VC_4_T_*x*_ in the acidic electrolyte confirmed a larger capacitance than in neutral or basic electrolytes. The GCD method was used to assess the cycling stability of Mo_4_VC_4_T_*x*_ in all four electrolytes. At a high current density of 10 A g^–1^, the Mo_4_VC_4_T_*x*_ film retained 84% (H_2_SO_4_, Figure 3d), 94% (LiCl, Figure S3a), 101% (Na_2_SO_4_, Figure S3b), and 109% (KOH, Figure S3c) of its initial capacitance after 8,000 cycles. In general, carbon-based electrode materials can retain a longer cycle life during prolonged charge–discharge cycling than pseudocapacitive materials, such as vanadium oxides^33^ due to their double-layer storage mechanism. The decrease in Mo_4_VC_4_T_*x*_ MXene capacitance can be associated with partially irreversible redox reactions.^28^ Moreover, the dissolution of Mo and V cations from Mo_4_VC_4_T_*x*_ MXene in acidic electrolytes during 8000 continuous charge–discharge cycles may result in a shorter cycle life compared to other media.^34^ It is important to note that the Mo_4_VC_4_T_*x*_ MXene exhibited a Coulombic efficiency of over 99.5% in all four electrolytes. Figure S3c,d shows that Mo_4_VC_4_T_*x*_ MXene exhibited enhanced capacitance with cycling in Na_2_SO_4_ and KOH. The slight improvement in capacitance can be attributed to increased accessibility of the inner layers of MXene electrodes.^15^ However, since the electrochemically accessible specific surface area of Mo_4_VC_4_T_*x*_ with the 1.3 nm thickness is smaller compared to MXenes with subnanometer thickness, such as V_2_CT_*x*_ or Mo_2_CT_*x*_, its specific capacitance is lower.

**Figure 4:**
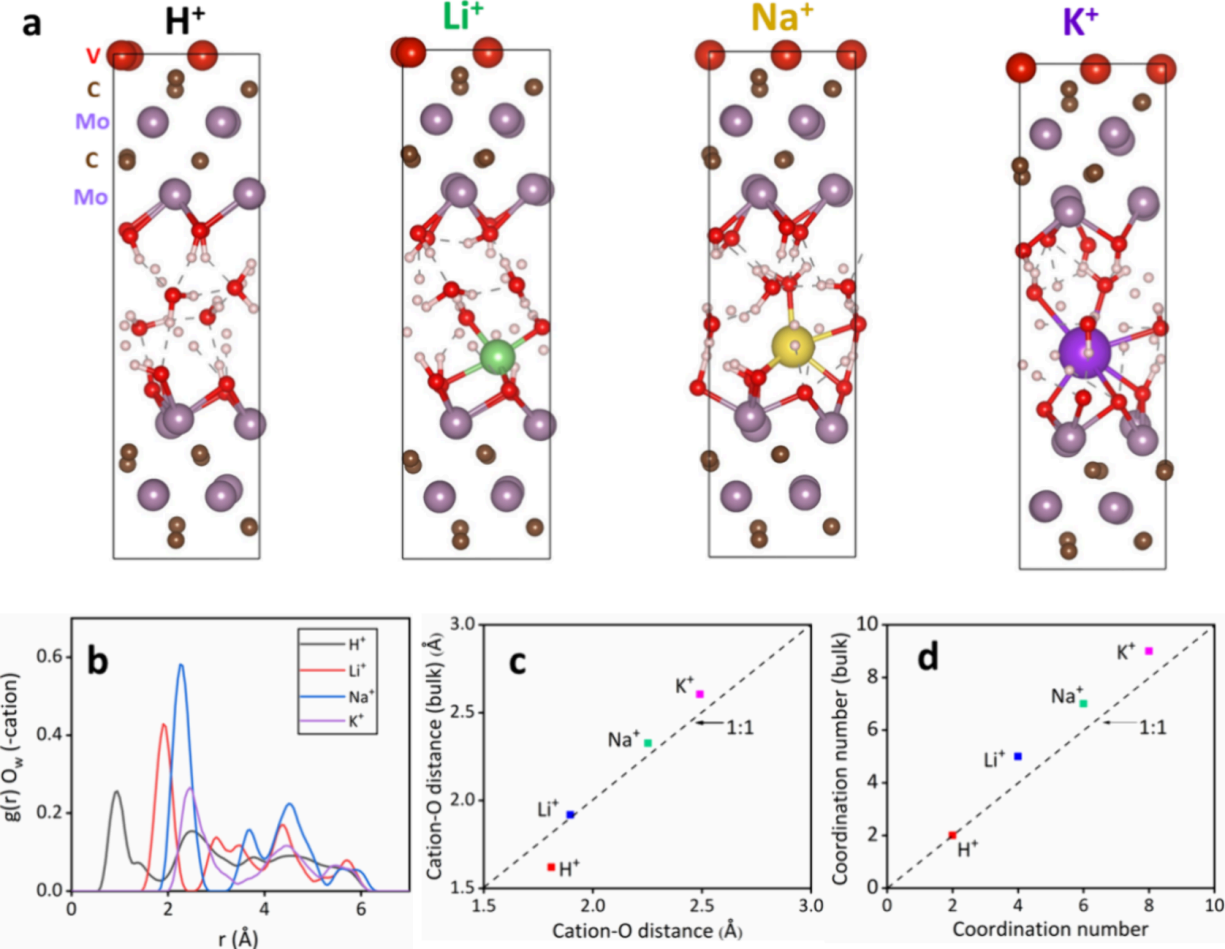
Cation arrangements in Mo_4_VC_4_T_*x*_ and their interactions with water via MD simulations: (a) Cation distributions in the interlayer space; (b) Radial distribution functions of oxygen in water, *g*(*r*), O_w_, around cations obtained from the AIMD modeling; (c) Cation–O_w_ distances corresponding to the positions of the maxima of *g*(*r*) demonstrate a clear correlation with values in bulk solutions; (d) Coordination numbers extracted from (b) in comparison with values for bulk solutions.

To better understand the charge storage mechanism, we investigated the intercalation states of four different monovalent cations (H^+^, Li^+^, Na^+^, and K^+^) representing the electrolytes and their interactions inside the MXene through AIMD simulations. The selected structural model consists of four Mo atoms on the surface, 12 water molecules, and a cation acting as an electrolyte. AIMD was chosen because of its high accuracy, which is required to characterize ion–water interactions. This method increases a nonempirical understanding of interactions in confined MXene layers and connects these interactions to the energetics and capacitive characteristics of the layers. It is known that the MXene surface is negatively charged,^35^ and thus, cations (H^+^, Li^+^, Na^+^, and K^+^) were considered for the calculations. The atomic structures of cations confined in the MXene layers are illustrated in Figure 4a. H^+^, Li^+^, Na^+^, and K^+^ demonstrated affinity toward the MXene surface and water molecules. Further, we utilized the radial distribution function, *g*(*r*), to determine the relative distance between the studied cations and oxygen in water (O_W_). The distance between a cation and oxygen was then calculated by determining the maximum radial distribution function *g*(*r*). According to Figure 4b, H^+^ showed the shortest cation–O_W_ distance, followed by Li^+^. This result indicates that H^+^ possessed the smallest hydration radii, and K^+^ showed the largest hydration radii in confinement, which is in agreement with previous studies.^36^ In addition, the first shell hydration radius was not influenced by such confinement, as demonstrated by the linear scaling of the distance between a cation and O_W_ in the bulk water system^36^ (Figure 4c). However, the coordination number of the confined cations was lower than that of the bulk system (Figure 4d). This result suggests that the studied cations underwent partial dehydration when the cations reached the narrow spaces of the MXene.

The PDOS of the simulated MXene structures in the presence of cations is shown in Figure S4. The specific computation approach and model simulation used were similar to those used in previous studies.^37,38^Figure S4a depicts the electronic distribution arising from the interaction between protons and oxygen functional groups. Based on the redox activity of MXene in the acidic electrolyte (Figures 3a,b), the pseudocapacitive behavior was caused by the reduction in the electrostatic potential difference during charge redistribution.^39^ Such pseudocapacitive behavior was accompanied by the appearance of highly reversible redox reactions (Figure S3b). Notably, for LiCl, NaOH, and KOH electrolyte systems, the adsorbed cations did not form bonds with functional groups. As a result, the contact between cations and the MXene surface was achieved through electrostatic attraction, leading to the manifestation of double-layer capacitance behavior. Theoretical calculations indicated that the adherence between the MXene surface and hydrated cation (Li^+^, K^+^, Na^+^) was inadequate (Figures S4b-d), leading to an EDLC behavior. The theoretical and experimental findings of this study suggest that Mo_4_VC_4_T_*x*_ exhibits pseudocapacitive redox behavior in H_2_SO_4_ and EDLC behavior in the LiCl, NaOH, and KOH electrolytes.

These experimental and theoretical results show that free-standing Mo_4_VC_4_T_*x*_ deserves further exploration in energy storage, conversion, and other electrochemical applications. First, it eliminates the need for binders, hence eliminating the addition of inactive materials. The prepared film can serve not only as a substrate but also as an active component of electrodes. Second, the thick MXene film can provide electrochemically active sites with various responses from different electrolytes. Through experimental and theoretical evaluations, pseudocapacitive behavior was observed for H_2_SO_4_, and EDLC behavior was found for the LiCl, NaOH, and KOH electrolytes. Further *in situ* characterization is necessary to investigate the dimensional and chemical changes inside electrodes and to completely understand the intercalation of ions and the electrochemical storage mechanism of Mo_4_VC_4_T_*x*_ in various electrolytes. Due to largely Mo surface coverage, Mo_4_VC_4_T_*x*_ has a narrow voltage window in acidic electrolytes and may be used as a catalyst or catalyst support for hydrogen evolution reaction (HER), similar to Mo_2_CT_*x*_.^40^ Dual-metal MXenes show promise in this application.^41^ However, if there is an expansion/contraction of the interlayer spacing during the insertion and withdrawal of ions, it can be used in electrochemical actuators, similar to Ti_3_C_2_T_*x*_. High rigidity of thicker M_5_C_4_ flakes may be advantageous. Energy harvesting applications should also be considered.^42,43^

## Conclusions

This study examined the electrochemical performance of free-standing Mo_4_VC_4_T_*x*_ electrodes, highlighting the effects of different aqueous electrolytes. At a scan rate of 2 mV s^–1^, the maximum gravimetric capacitance values obtained in 1 M H_2_SO_4_, 5 M LiCl, 3 M Na_2_SO_4_, and 3 M KOH were 219, 98, 69, and 66 F g^–1^, respectively. Satisfactory capacitance retention of Mo_4_VC_4_T_*x*_ film was found in H_2_SO_4_ (84%) and LiCl (94%) electrolytes after 8000 cycles. Stable electrochemical performance was observed in neutral (101% in Na_2_SO_4_) and basic (109% in KOH) electrolytes. A redox process was observed in 3 M H_2_SO_4_ electrolyte, which resulted from a strong interaction between hydronium ions (protons) and oxygen-containing functional groups and accounted for higher capacitance than that of the other systems. The intercalation states of four different cations (H^+^, Li^+^, Na^+^, and K^+^) inside the MXene were studied through AIMD simulations and DFT calculations. Overall, this study provides the foundation for the exploration of Mo_4_VC_4_T_*x*_ in energy storage and other electrochemical applications.

## Supporting Information Available

Experimental details, structural and compositional characterization, electrochemical properties, computational details, CV curves at different scan rates, GCD curves at various current densities, comparison of cycling stability, and DFT calculations (PDF)
