# Supplementary material for: Electrochemical Properties of Mo4VC4Tx MXene in Aqueous Electrolytes
Source: ACS Appl Mater Interfaces. 2024 Jul 15;16(29):38053–60. doi: 10.1021/acsami.4c06519 (PMC11284742; doi:10.1021/acsami.4c06519)
Supplement: Supplementary file 1 — am4c06519_si_001.pdf [file am4c06519_si_001.pdf]

Supporting information

Electrochemical Properties of $Mo_4VC_4T_x$ MXene in Aqueous Electrolytes

Iftikhar Hussain [a, b], Faisal Rehman [c,d,e], Mohit Saraf [b], Teng Zhang [b], Ruocun Wang [b], Tridip Das[d], Zhengtang Luo [c], Yury Gogotsi [b*], Kaili Zhang [a*]

[a] Department of Mechanical Engineering, City University of Hong Kong, 83 Tat Chee Avenue, Kowloon 999077, Hong Kong

[b] A.J. Drexel Nanomaterials Institute and Department of Materials Science and Engineering, Drexel University, Philadelphia, Pennsylvania 19104, United States

[c] Department of Chemical and Biological Engineering, The Hong Kong University of Science and Technology, Clear Water Bay, Kowloon 999077, Hong Kong

[d] Materials and Process Simulation Center (MSC), MC 139-74, California Institute of Technology, Pasadena, CA 91125, USA

[e] Department of Chemical & Polymer Engineering, University of Engineering & Technology Lahore, Faisalabad Campus, 3.5km, Khurrianwala – Makkuana By-Pass, Faisalabad 38000, Pakistan

*Corresponding authors: gogotsi@drexel.edu (Prof. Yury Gogotsi)

kaizhang@cityu.edu.hk (Prof. Kaili Zhang)

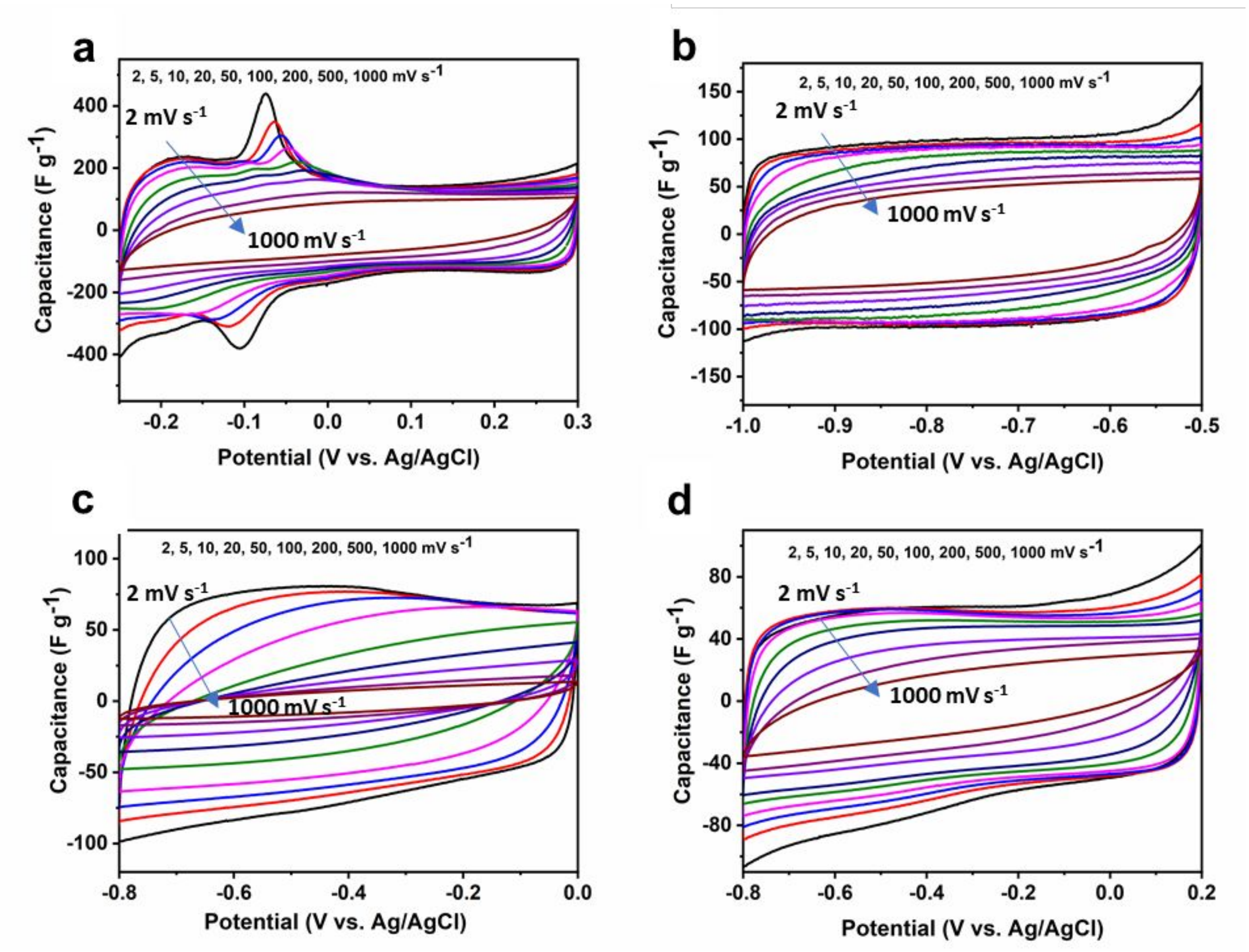

Figure S1. CV curves at different scan rates ranging from 2 to 1000 mV s^{-1} in (a) 3 M H_2SO_4, (b) 3 M KOH, (c) 5 M LiCl, and (d) 1 M Na_2SO_4.

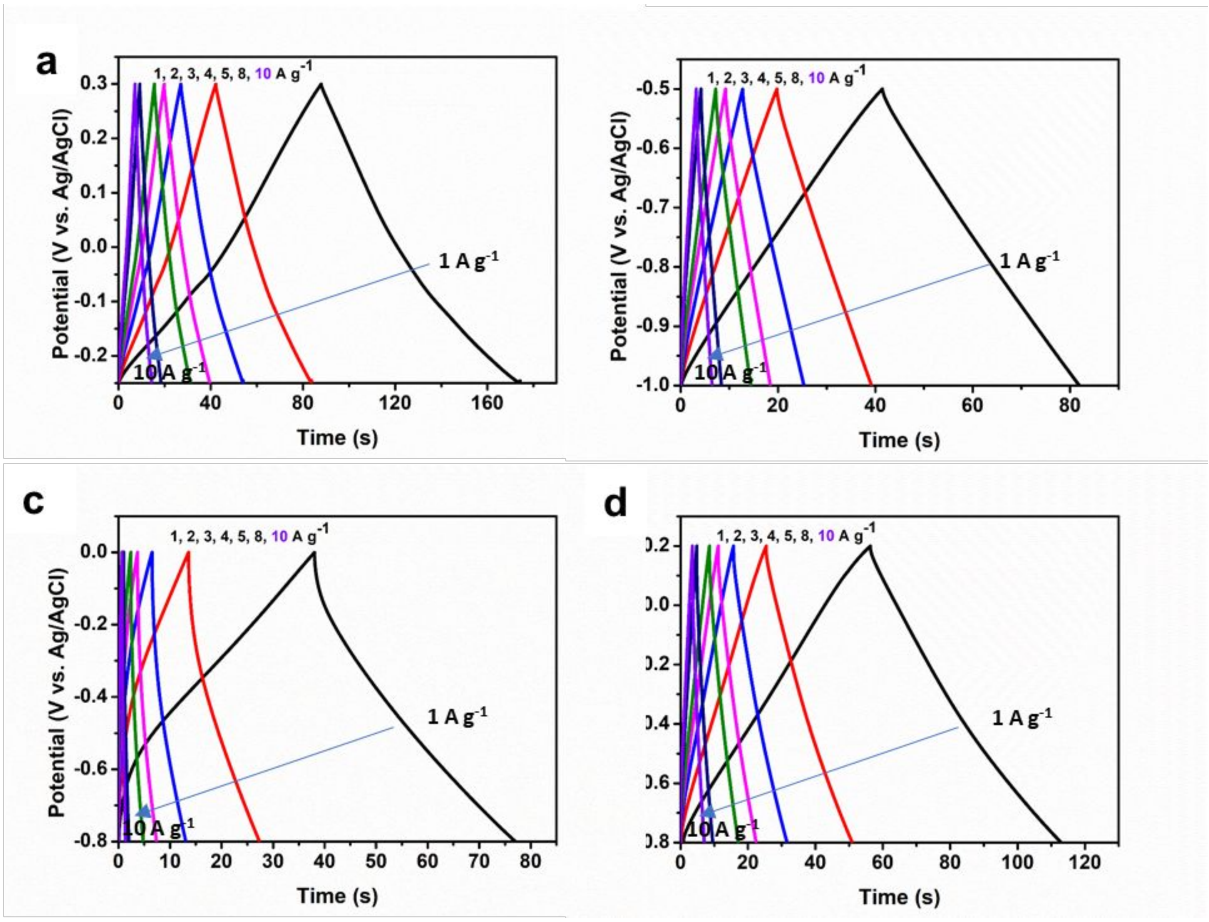

Figure S2. GCD curves at different current densities ranging from 1 to 10 A g^{-1} in (a) 3 M H_2SO_4, (b) 3 M KOH, (c) 5 M LiCl, and (d) 1 M Na_2SO_4.

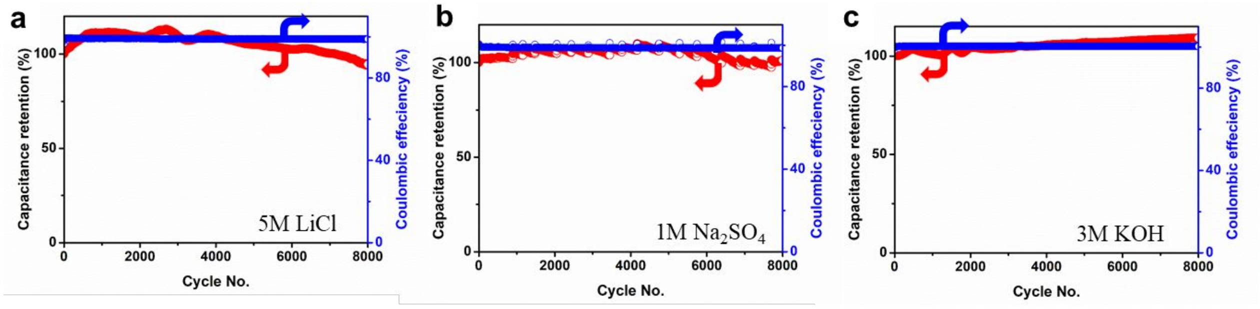

Figure S3. Cycling stability testing of the delaminated $Mo_4VC_4T_x$ film in (a) 5 M LiCl, (b) 1 M Na_2SO_4, and (c) 3 M KOH for 8000 cycles.

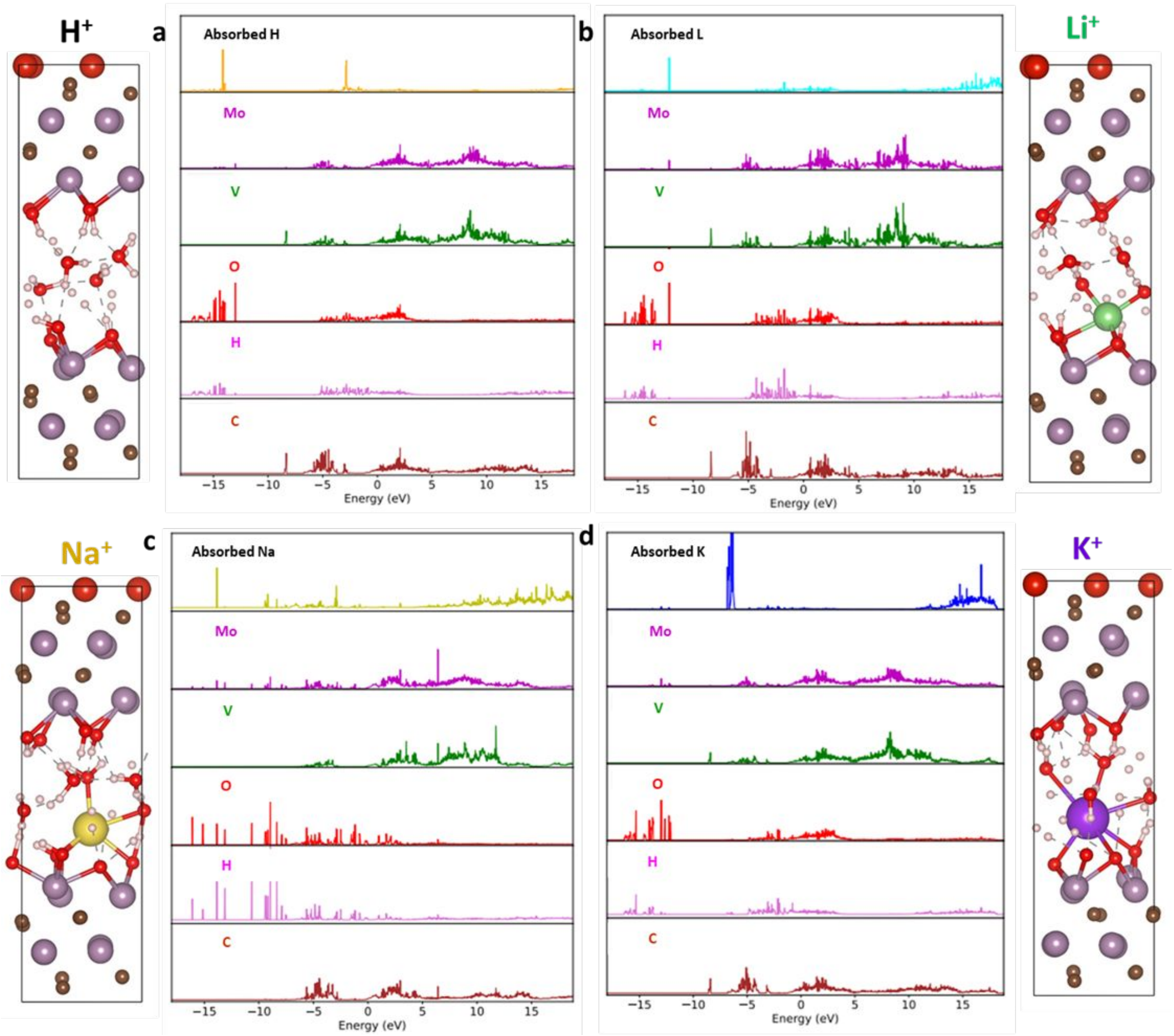

Figure S4. Simulated model of $Mo_4VC_4T_x$ MXene and PDOS of absorbed atoms of (a) H in H_2SO_4 electrolyte, (b) Li in LiCl electrolyte, (c) Na in NaOH electrolyte, and (d) K in KOH electrolyte.
